# Painful, degenerating intervertebral discs up-regulate neurite sprouting and CGRP through nociceptive factors

**DOI:** 10.1111/jcmm.12268

**Published:** 2014-03-20

**Authors:** Emerson Krock, Derek H Rosenzweig, Anne-Julie Chabot-Doré, Peter Jarzem, Michael H Weber, Jean A Ouellet, Laura S Stone, Lisbet Haglund

**Affiliations:** a Orthopeadic Research Laboratory, Division of Orthopedic Surgery, McGill UniversityMontreal, QC, Canada; b McGill Scoliosis and Spine Research GroupMontreal, QC, Canada; c Alan Edwards Centre for Research on PainMontreal, QC, Canada; d Integrated Program in Neuroscience, McGill UniversityMontreal, QC, Canada; e Department of Anesthesiology, McGill UniversityMontreal, QC, Canada; f Department of Pharmacology and Therapeutics, Faculty of Medicine, McGill UniversityMontreal, QC, Canada; g Department of Faculty of Dentistry, McGill UniversityMontreal, QC, Canada

**Keywords:** intervertebral disc degeneration, discogenic pain, human, nerve growth factor, CGRP, inflammatory cytokines

## Abstract

Intervertebral disc degeneration (IVD) can result in chronic low back pain, a common cause of morbidity and disability. Inflammation has been associated with IVD degeneration, however the relationship between inflammatory factors and chronic low back pain remains unclear. Furthermore, increased levels of nerve growth factor (NGF) and brain derived neurotrophic factor (BDNF) are both associated with inflammation and chronic low back pain, but whether degenerating discs release sufficient concentrations of factors that induce nociceptor plasticity remains unclear. Degenerating IVDs from low back pain patients and healthy, painless IVDs from human organ donors were cultured *ex vivo*. Inflammatory and nociceptive factors released by IVDs into culture media were quantified by enzyme-linked immunosorbent assays and protein arrays. The ability of factors released to induce neurite growth and nociceptive neuropeptide production was investigated. Degenerating discs release increased levels of tumour necrosis factor-α, interleukin-1β, NGF and BDNF. Factors released by degenerating IVDs increased neurite growth and calcitonin gene-related peptide expression, both of which were blocked by anti-NGF treatment. Furthermore, protein arrays found increased levels of 20 inflammatory factors, many of which have nociceptive effects. Our results demonstrate that degenerating and painful human IVDs release increased levels of NGF, inflammatory and nociceptive factors *ex vivo* that induce neuronal plasticity and may actively diffuse to induce neo-innervation and pain *in vivo*.

## Introduction

Low back pain has a lifetime prevalence of 60–80% and is associated with profound socioeconomic costs [1]. Intervertebral disc (IVD) degeneration is a major cause of low back pain [2]. The IVD is composed of two distinct regions; the outer annulus fibrosus (AF) and the central nucleus pulposus (NP). Healthy, pain-free IVDs are mostly avascular and aneural with neurites penetrating only the outer layers of the AF. However, evidence suggests that degenerating, painful IVDs are innervated [3], supporting a relationship between discogenic pain and increased IVD innervation. The extracellular matrix of healthy IVDs contains high concentrations of negatively charged proteoglycans, providing an unfavourable environment for neurite growth [4]. However, with degeneration proteoglycans are fragmented and released from the tissue [2], potentially creating an environment more permissive to neurite ingrowth.

Several animal models for disc degeneration show increased intervertebral disc innervation by the identification of calcitonin gene-related peptide (CGRP) and Substance P-expressing fibres [5–7], the majority of which are thought to be nociceptors. Nociceptive fibres have also been reported in degenerate human disc tissue [3,8,9], suggesting a mechanistic role in pain associated with degenerative disc disease. However, the direct mechanism of discogenic pain *in vivo* has yet to be established.

Nerve damage and neuronal sensitization are hypothesized to play a role in chronic pain associated with degenerative disc disease [10,11]. As degenerating IVDs lose height or herniate, the dorsal root ganglion (DRG), nerve root or spinal cord can be compressed, leading to neuropathic pain [12,13]. Furthermore, the inflammatory processes involved in IVD degeneration have been hypothesized to contribute to chronic back pain [12]. These factors can induce neuronal sensitization, leading to the development of inflammatory pain, a process separate from neuropathic pain. *In vitro* studies on either treated disc cells or cells isolated from degenerating IVDs have shown increases in pro-inflammatory and pro-nociceptive factors such as interleukins IL-1β, IL-6, tumour necrosis factor-α (TNF-α), nerve growth factor (NGF) and brain derived neurotrophic factor (BDNF) [12,14,15]. NGF and BDNF are neurotrophins that promote neuronal development, survival and growth. NGF and BDNF produced *in vitro* by cultured disc cells stimulate neurite growth in neuronal cell lines [16]. They can also modulate pain and increase the expression of the nociceptive neuropeptides Substance P and CGRP in neurons [10]. Therefore inflammatory and neurotrophic factors may play a significant role in discogenic pain. However, it remains unclear whether degenerating human IVDs release sufficient concentrations of NGF and BDNF to stimulate neurite growth and nociceptive peptide production.

The present study investigates the factors actively released by degenerating and painful IVDs, and determines whether they can directly induce innervation and activation of pain-sensing fibres. We show in real-time that *ex vivo,* degenerating IVDs release factors that induce neurite growth and nociceptor plasticity, as compared to healthy disc cultures. From this study, we propose that inflammation associated with IVD degeneration may directly contribute to the development of chronic low back pain.

## Materials and methods

### Tissue sources

This study was approved by McGill University Institutional Review Board (IRB# A04-M53-08B) project titled ‘Human Intervertebral Discs used for Culture and Extracellular Matrix’. Eight degenerating IVDs from six females and two males, ages 33–58 years (mean 40.4 years) were resected en bloc from consenting patients undergoing discectomy, interbody arthroplasty or fusion for chronic discogenic axial low back pain. Throughout this study, these surgically removed samples are called degenerating, painful IVDs. Eleven healthy, pain-free IVDs from six female and two male organ donors, ages 20–50 years (mean 35.6 years) were obtained through the Transplant Quebec Organ Donation Program from individuals who had undergone sustained brain death. IVDs from organ donors were inspected for visual signs of degeneration. X-rays of the lumbar spinal segments were evaluated for signs of disc degeneration, loss of disc height, endplate spurs and intradiscal calcification. In addition, a family member completed a back pain questionnaire about pain history and treatment for back pain of the donor. Intervertebral discs that showed signs of degeneration or came from donors with a history of back pain were excluded from the study.

### IVD isolation and culture

Healthy control IVDs were isolated from organ donors as previously described [17]. Briefly, both degenerating and healthy IVDs were cultured using a method that has more than 95% cell viability after 7 days in culture [17,18]. Briefly, intact discs were washed in PBS supplemented with 5 μg/ml Gentamicin (Gibco, Burlington, ON, Canada) and 0.125 μg/ml fungizone (Gibco) for 5 min., then twice in Hanks Balanced Salt Solution (HBSS; Sigma-Aldrich, St. Louis, MO, USA) supplemented with 5 μg/ml Gentamicin (Gibco) and 125 ng/ml fungizone (Gibco) for 5 min. Discs were cultured in 3.5 ml per gram of tissue in serum-free IVD media (DMEM, Sigma-Aldrich), 50 μg/ml ascorbic acid, 5 μg/ml Gentamicin (Gibco), 0.125 μg/ml fungizone (Gibco) and 1× glutamax (Gibco) as previously described [17,18]. A volume of 3.5 ml media per gram of tissue is sufficient to completely submerge discs and to maintain long-term cell viability [17,18]. Cultures were maintained at 37°C and 5% CO_2_ for 48 hrs. Degenerating, painful IVD conditioned media and healthy, pain-free IVD conditioned media were collected and frozen as individual samples at −80°C for later analysis. The conditioned medium was filter sterilized and supplemented with 5 μg/ml Gentamicin (Gibco), penstrep (25 U/ml Penicillin, 25 μg/ml Streptomycin, Gibco) and 0.125 μg/ml fungizone (Gibco) prior to use in neuronal cultures.

### Conditioned media analysis

The concentration of TNF-α (ELH-TNFALPHA-001; RayBiotech, Norcross, GA, USA), NGF (ELH-BNGF-001; RayBiotech), BDNF (ELH-BDNF-001; RayBiotech) and IL-1β (ELH-IL1BETA-001; RayBiotech) in the culture medium was quantified using ELISA according to manufacturers' instructions. Duplicate 100 μl samples of each conditioned medium were incubated in ELISA plates overnight at 4°C. Colorimetric absorbance was measured with a Tecan Infinite M200 PRO (Tecan, Männedorf, Switzerland) and analysed with i-control 1.9 software (Tecan). Duplicates were averaged and the mean concentrations for healthy and degenerating IVDs were calculated.

RayBio Human Cytokine Array 1 Maps (product code: AAH-CYT-1; RayBiotech Inc.) were used to determine the relative quantities of 23 cytokines according to manufacturer's instructions. Arrays were imaged with the provided enhanced chemiluminescence kit using an ImageQuant LAS4000 (GE Healthcare, Baie d'Urfe, QC, Canada). ImageQuant TL array analysis software (GE Healthcare) was used to analyse the blots. The relative quantity of each factor present in each media sample was calculated using the controls included on the protein arrays. Mean relative quantities of each factor for the degenerating and healthy groups were then calculated.

### Mouse DRG neuron isolation

Studies were approved by the Animal Care Committee at McGill University, and conformed to the ethical guidelines of the Canadian Council on Animal Care and the Committee for Research and Ethical Issues of IASP. Mouse DRG cell cultures were derived as previously described by Malin *et al*. [19]. Dorsal root ganglion were dissected from the spine, digested in papain (Worthington Biochemical Corporation, Lakewood, NJ, USA), followed by collagenase type II (Worthington). Cells were mechanically dissociated by pipetting the solution up and down, the suspension was then passed through a cell strainer to separate the neurons from remaining debris. The neurons were collected by centrifugation and resuspended in F12 medium (Gibco) supplemented with 10% FBS, 10 U/ml Penicillin and 10 μg/ml Streptomycin (Gibco). The isolated neurons were cultured in eight well chamber slides (BD Biosciences, Bedford, MA, USA) coated with 5 μg/ml Laminin (BD Biosciences) and 5 μg/ml Poly-d-Lysine (BD Biosciences) at 37°C and 5% CO_2_.

### Cell culture

Rat adrenal pheochromocytoma (PC12) cell line expresses the receptor for and responds to NGF. When exposed to NGF they take on a neuronal-like phenotype. They are commonly used to study neuronal differentiation and neurite sprouting [20–22]. PC12 cells (ATCC, Manassas, VA, USA) in passages 2–7 were cultured on six-well plates (Nunc) or eight-well chamber slides (Nunc) coated with 50 μg/ml rat tail collagen type I (Gibco) and 10 μg/ml Poly-l-Lysine (Sigma-Aldrich). The cells were maintained for 24 hrs in RPMI (Gibco) media containing 10% horse serum (Gibco), 5% FBS (Gibco) and 1× antibiotic/antimycotic (anti-anti) solution (Gibco).

PC12 and neuronal culture media were replaced after a 24 hr acclimatization period, with IVD medium supplemented with 0.1% FBS (Gibco) containing either no NGF, 2.5 ng/ml (Bioshop, Burlington, ON, Canada; PC12 cells), 100 pg/ml (PC12 cells and neurons) or 10 ng/ml NGF (neurons), degenerating IVD conditioned medium or healthy IVD conditioned medium. 2.5 ng/ml NGF for PC12 cells was selected as the positive control based on a serial dilution that showed an effect similar to higher doses of NGF (data not shown). 10 ng/ml NGF was selected for neuronal cultures based on the literature [23]. PC12 cells were exposed for 24 or 48 hrs to the different media (*n* = 3 in each IVD media group, *n* = 2 for each control for 24 hr cultures and *n* = 6 in each IVD media group, *n* = 3 for each control for 48 hr cultures). Dorsal root ganglion neurons were exposed for 48 hrs to the different media (*n* = 3 in each IVD media group, *n* = 2 for each control group) for conditioned media cultures, samples from the low, middle and high range of NGF concentrations were used.

Nerve growth factor neutralization experiments were performed over 48 hrs using a mouse monoclonal NGF antibody (Exalpha Biologicals Inc., Shirley, MA, USA) raised against human NGF. A 200-fold molar excess compared to 10 ng/ml was used. The antibody was pre-incubated with media containing 10 ng/ml and 100 pg/ml NGF (*n* = 2 for each) and IVD conditioned media (*n* = 3 for PC12 cells, *n* = 2 for neurons) for 1 hr at room temperature prior to applying to neuronal cultures. Anti-NGF was added to media with NGF to ensure antibody efficacy. Pre-immune mouse IgG (Sigma-Aldrich) used at the same concentration as NGF antibody was incubated with NGF containing media prior to application to cultures.

### PC12 culture image acquisition and neurite analysis

Each medium was applied in duplicate wells and two random images per well were taken using a Zeiss Axiovert 40 C inverted light microscope (Toronto, ON, Canada) with a Canon PowerShot A640 camera and 52 mm Soligor adaptor tube (Mississauga, ON, Canada). The percentage of cells with neurites was determined and then averaged for each experimental condition.

### Reverse transcription and quantitative real-time PCR

Quantitative real-time PCR (qRT-PCR) was performed after 24 and 48 hrs on the same cultures used to quantify neurite growth (*n* = 3 in each IVD media group, *n* = 2 for each control for 24 hr cultures and *n* = 6 in each IVD media group, *n* = 3 for each control for 48 hr cultures). RNA was extracted from PC12 cultures using TRIzol Reagent (Invitrogen, Burlington, On, Canada). Approximately 500 ng of RNA was reverse transcribed to cDNA (qSqript cDNA; Quanta Biosciences, Gaithersburg, MD, USA) using an Applied Biosystem Veriti thermal cycler (Applied Biosystems, Carlsbad, CA, USA). qRT-PCR was performed as previously described [24]. Briefly, qRT-PCR was performed with PerfeCTa SYBR Green FastMix (Quanta Biosciences) on an Applied Biosystems StepOnePlus using specific primers [25] to Neurofilament Light Chain (NF-L), plasminogen activator, urokinase receptor (Plaur), polo-like kinase 2 (Plk2) poliovirus receptor (PVR), vaccinia growth factor (VGF), which are associated with neurite growth [25]. β-actin was used as an endogenous control and average fold change in each gene was normalized to the no NGF control according to the 2^−ΔΔCt^ method [26].

### Immunohistochemistry and image acquisition of DRG cultures

Dorsal root ganglion cultures were fixed for 10 min. in 4% paraformaldehyde at room temperature, washed three times in PBS and incubated at room temperature for 1 hr in blocking buffer containing 0.3% Triton X-100, 1% bovine albumin serum, 1% normal donkey serum, 0.1% sodium azide in PBS. The slides were then incubated with a Protein Gene Product (PGP 9.5) rabbit monoclonal antibody (1:2000; Ultraclone Limited, Isle of Wight, UK, catalogue number RAB95101) and a CGRP sheep polyclonal antibody (1:1000; Enzo Life Sciences Inc., Farmingdale, NY, USA, catalogue number CA1137, lot 12031227) in blocking buffer overnight at 4°C. Slides were washed three times in PBS and incubated with the secondary antibodies (Alexa Flour® 488-conjugated Donkey anti-rabbit, catalogue number 711-545-152, and Cy™3-conjugated Donkey Anti-Sheep, product number 713-165-147, Jackson ImmunoResearch Laboratories Inc., West Grove, PA, USA) for 1.5 hrs at room temperature followed by exposure to DAPI (1:5000; Sigma-Aldrich) in PBS for 10 min. and then two washes with PBS. Slides were mounted using Aqua Polymount (Polysciences Inc., Warrington, PA, USA) and images were acquired using an Olympus BX51 (Tokyo, Japan) microscope equipped with a colour digital camera (Olympus DP71). Five random images (20× magnification) were taken of each well, making a total of 10 images per condition tested. A total of 20 images of each control were taken and 30 images of degenerating and 30 images of healthy media-treated cells were taken. For anti-NGF neutralization experiments, 10 images per control condition and 20 images per degenerating media-treated cells were taken.

### DRG culture image analysis

The acquired images of each location were combined in Photoshop CS2 and ImageJ was used to establish thresholds for positive staining of each marker. PGP 9.5 (green)- and CGRP (red)-immunoreactive cells were counted in separate channels. Images were assessed in a blinded and randomized manner. Calcitonin gene-related peptide immunoreactive neurons are reported as a percentage of all neurons (PGP 9.5-immunoreactive cell bodies). All CGRP-immunoreactive cell bodies were also PGP 9.5 immunoreactive.

### Statistical analysis

Graphpad Prism 6 (La Jolla, CA, USA) was used for all statistical analyses. Unpaired *t*-tests were used to test for differences between degenerative and healthy groups for each factor present on antibody arrays and TNF-α and NGF ELISAs. Unpaired Mann–Whitney test was used for BDNF ELISA analysis. Differences between neurite sprouting and CGRP expression were tested using one-way anovas with *post hoc* Tukey tests. Gene expression from 2.5 ng/ml NGF, healthy IVD media and degenerating IVD media cultures was compared to the –NGF control using a two-tailed *t*-test. For all tests, significance was established at *P* < 0.05. All data are presented as the mean value ± standard error of the mean in the text. Data are graphed as mean values with the 95% confidence intervals.

## Results

### Degenerating IVDs release increased quantities of TNF-α, IL-1β, NGF and BDNF

**Figure 1:**
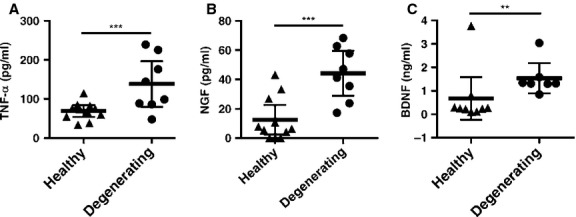
Tumour necrosis factor-α (TNF-α; A), nerve growth factor (NGF; B) and brain derived neurotrophic factor (BDNF; C) mean concentrations in media from healthy pain-free or degenerating, painful intervertebral disc (IVDs). *n* = 8 in degenerate, painful group and *n* = 11 in healthy, pain-free group for TNF-α and NGF ±95% CI, unpaired *t*-test. *n* = 7 in degenerating, painful group and *n* = 9 in healthy, pain-free group for BDNF, ±95% CI, Mann–Whitney test. **P < 0.01, ***P < 0.001.

As isolated IVD cells can release inflammatory and nuerotrophic factors [27,28] and histological analyses of degenerate tissue have confirmed this [8,29], we quantified the ability of degenerating IVDs to actively release these factors *ex vivo* into culture media. Intervertebral discs was cultured on a volume per weight. ELISA analysis revealed that degenerating, painful IVDs released a significantly greater amount of TNF-α (138.3 ± 24.7 pg/ml, *P* = 0.01, Fig.1A) compared to healthy, pain-free IVDs (69.2 ± 22.6 pg/ml). NGF released at a significantly greater amount by degenerating, painful IVDs (44.2 ± 6.5 pg/ml, *P* < 0.001, Fig.1B) compared to healthy, pain-free IVDs (12.5 ± 4.5 pg/ml). Brain derived neurotrophic factor was significantly higher in the media from degenerating, painful IVDs (1.54 ± 0.026 ng/ml, *P* = 0.004, Fig.1C), compared to control IVDs (0.67 ± 0.039 ng/ml). Degenerating, painful IVDs released detectable amounts of IL-1β, but not all healthy, pain-free IVDs released IL-1β above the detection limit (0.48 pg/ml) of the assay (data not shown). Therefore, the difference between the two groups was not determined.

### Degenerating, painful IVD conditioned media increase neurite sprouting in PC12 cells

**Figure 2:**
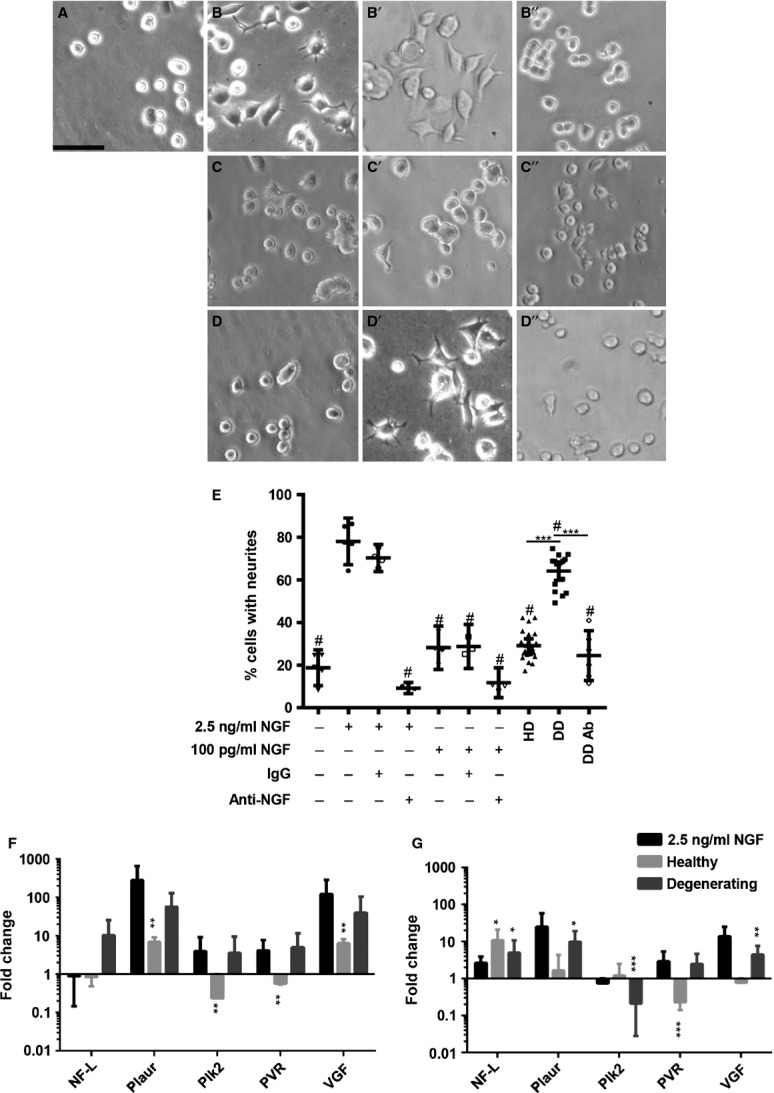
PC12 neurite growth after 48 hrs of culture. Representative phase contrast image of untreated cultures (A), cultures treated with 2.5 ng/ml nerve growth factor (NGF; B), 2.5 ng/ml NGF and normal IgG (B'), 2.5 ng/ml NGF and anti-NGF antibody (B”), 100 pg/ml NGF (C), 100 pg/ml NGF and normal IgG (C'), 100 pg/ml NGF and anti-NGF antibody (C”), healthy pain-free intervertebral disc (IVD) media conditioned media (D), degenerating, painful IVD conditioned media (D') and degenerating, painful IVD conditioned media treated with anti-NGF antibody (D”). Scale bar: 62.5 μm. (E) Quantification of the proportion of cells with neurites after 48 hrs of culture. Untreated cultures, cultures with 2.5 ng/ml NGF, 100 pg/ml NGF, IgG and anti-NGF antibodies in different combinations were quantified as indicated. Neurite sprouting in Control cultures healthy pain-free IVD media (HD) cultures, degenerating, painful IVD media cultures DD and degenerating, painful IVD media cultures treated with anti-NGF (DD Ab) were quantified as indicated. Fold changes of marker genes compared to –NGF control for PC12 neuronal differentiation and growth measured by qRT-PCR after 24 hrs (F) and 48 hrs (G). *n* = 3 in each IVD media group, *n* = 2 for each control in 24 hr cultures. *n* = 6 in each IVD media group, *n* = 3 for each control for 48 hr cultures. *n* = 3 for DD Ab and *n* = 2 for 2.5 ng/ml and 100 pg/ml NGF and NGF IgG. Error bars; ±95% CI, one-way anova. *P < 0.05, **P < 0.01, ***P < 0.001. #*P* < 0.001 when compared to 2.5 ng/ml NGF control.

As degenerating, painful IVDs release increased amounts of NGF and BDNF *ex vivo*, the conditioned media were tested for the ability to stimulate neurite growth in PC12 cells. After 48 hrs of culture, 20 ± 3% of untreated cells, 78 ± 4% of NGF-treated cells, 29 ± 2% of cells cultured in healthy, pain-free IVD media and 64 ± 2% of cells cultured in degenerating, painful IVDs had neurites (Fig.2A and B; *n* = 6 IVDs for each conditioned media group). A significantly greater proportion of cells treated with NGF extended neurites when compared to untreated cells (*P* < 0.0001). There was no significant difference in the proportion of cells with neurites between untreated and healthy IVD media groups (*P* = 0.25). NGF cultures had a greater proportion of cells compared to degenerating media groups (*P* < 0.01). However, degenerating, painful IVD media induced neurite sprouting in a significantly greater percentage of cells compared to cells cultured in healthy, pain-free IVD media (*P* = <0.0001) (Fig.2) indicating that painful, degenerating IVDs produce factors that promote neurite growth.

The ability of NGF released by degenerating IVDs to induce neurite sprouting was determined through NGF sequestration. In these experiments, 100 pg/ml NGF was used as an additional control because this is similar to the highest concentration measured in degenerating IVD conditioned media (Fig1B). However, 100 pg/ml NGF alone was not sufficient to drive a significant increase in neurite sprouting in 48 hrs compared to untreated cultures (28 ± 3% *versus* 19 ± 3%, *P* = 0.667). Anti-NGF treatment significantly reduced the percentage of cells with neurites (9 ± 1%) in cultures treated with 2.5 ng/ml NGF (*P* < 0.001), whereas normal IgG had no effect on neurite sprouting (70 ± 2%, *P* = 0.8517). Anti-NGF treatment of degenerating, painful IVD media significantly reduced the percentage of cells with neurites compared to degenerating IVD media without antibody (25 ± 5% *versus* 64 ± 2%, *P* < 0.001, *n* = 3 IVD samples for each group). There was no significant difference in the percentage of cells with neurites between healthy IVD media and anti-NGF-treated degenerating media cultures (*P* = 0.929, Fig.2). Interestingly, this data indicate that NGF released by degenerating IVDs is required for increased neurite growth but that additional factors are required to allow such low concentrations to stimulate neurite growth.

### Gene expression associated with neurite growth is increased in degenerating, painful IVD media cultures

To confirm that degenerating, painful IVD media induce neuronal differentiation, gene expression analysis of common neuronal markers was performed. After 24 hrs of culture, degenerating, painful IVD media caused strong trends for increased expression of NF-L, Plaur, Plk2, PVR and VGF (Fig.2F). Healthy media caused both up- and down-regulation of gene expression. At 48 hrs, degenerating IVD media caused a significant up-regulation of NF-L, Plaur and VGF, whereas healthy media caused a significant up-regulation of only NF-L.

### Degenerating, painful IVD media increase CGRP expression in mouse DRG neurons

**Figure 3:**
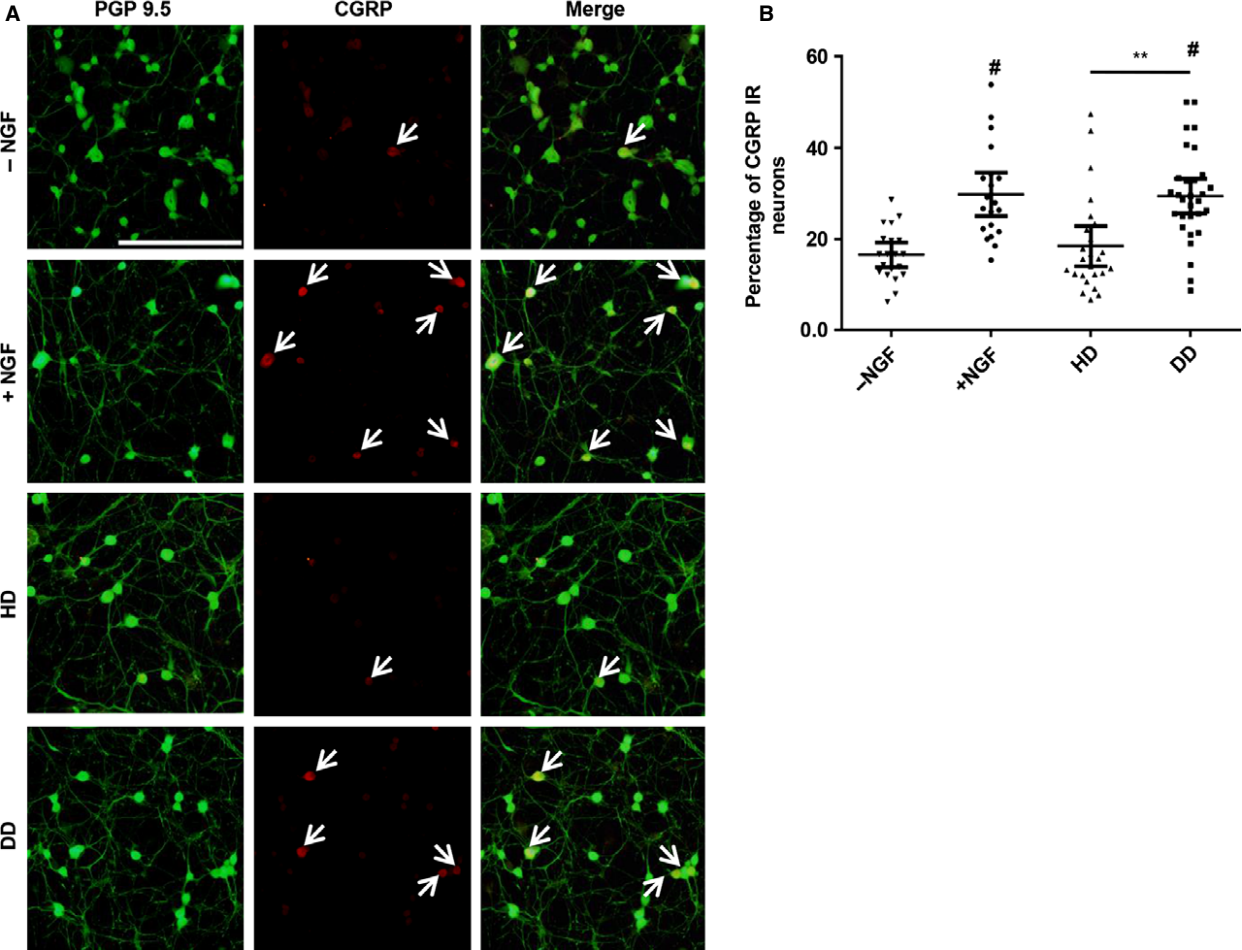
Calcitonin gene-related peptide (CGRP) immunoreactivity in mouse dorsal root ganglion neurons after 48 hrs of culture. (A) Representative fluorescent images of neuronal cultures that were untreated (row 1), treated with 10 ng/ml nerve growth factor (NGF; row 2), maintained in healthy, pain-free intervertebral disc (IVD) conditioned media (row 3) or in degenerating, painful IVD media (row 4). PGP 9.5 (green) is a general neuronal marker and CGRP (red) is nociceptive neuropeptide. PGP 9.5 and CGRP are overlaid in merged images. White arrows indicate CGRP-immunoreactive neurons; scale bar: 200 μm. (B) Quantification of CGRP immunoreactivity for each group. −NGF; untreated media, +NGF; media supplemented with NGF, HD; healthy disc conditioned media, DD, degenerating, painful IVD conditioned media. *n* = 3 samples per group, tested in duplicate, with five fields counted per duplicate totalling 10 fields counted per condition, error bars; ±95% CI, one-way anova. **P < 0.01. #*P* < 0.001 when compared to −NGF control.

Calcitonin gene-related peptide is a neurotransmitter that acts as a pain modulator and increased production can cause hyperexcitability and sensitization. To determine the effect of degenerating, painful IVD media on CGRP expression, DRG neurons were exposed to this media and compared healthy pain-free IVD media. After 48 hrs, neuronal cultures were analysed for the expression of the general neuronal marker PGP 9.5 (green) and CGRP (red; Fig.3A). The proportion of neurons that showed CGRP expression was analysed for each treatment. 16 ± 1% of untreated neurons, and 30 ± 2% of 10 ng/ml NGF-treated neurons expressed CGRP. Similar to untreated controls, 18 ± 2% of neurons cultured in healthy, pain-free IVD media expressed CGRP. Similar to NGF-treated controls, 29 ± 2% of neurons cultured in degenerating, painful IVD media were CGRP immunoreactive. 10 ng/ml NGF-treated neurons had a significantly higher percentage of CGRP-immunoreactive cells compared to untreated controls (*P* = 0.0045). There was no difference in CGRP immunoreactivity between non-treated and healthy, pain-free media groups (*P* = 0.9999) or between NGF-treated and degenerating, painful media groups (*P* > 0.9999). A significantly greater proportion of cells cultured in degenerating, painful IVD were CGRP immunoreactive compared to neurons cultured in healthy, pain-free IVD media (*P* = 0.007, Fig.3B).

**Figure 4:**
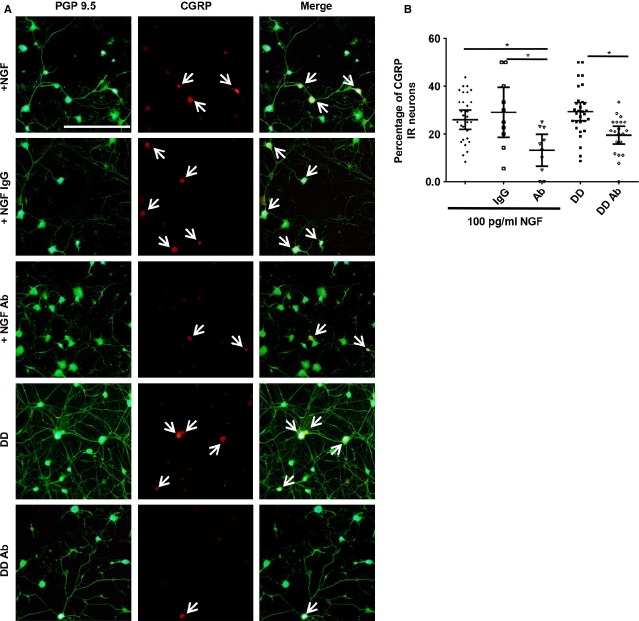
Calcitonin gene-related peptide (CGRP) expression in mouse dorsal root ganglion neurons after 48 hrs of culture. (A) Representative fluorescent images of neuronal cultures treated with 100 pg/ml nerve growth factor (NGF; +NGF, Row 1), 100 pg/ml NGF and normal IgG (+NGF IgG, Row 2), 100 pg/ml NGF and anti-NGF antibody (+NGF Ab, Row 3), media conditioned by degenerating, painful intervertebral disc (IVDs; DD, Row 4), or degenerating IVD media with anti-NGF antibody (DD Ab, Row 5). PGP 9.5 (green) is a general neuronal marker and CGRP (red) is pain neurotransmitter. PGP 9.5 and CGRP are overlaid in merged images. White arrows indicate CGRP-immunoreactive neurons; scale bar: 200 μm. (B) Quantification of CGRP immunoreactivity for each group. *n* = 2 samples per group, tested in duplicate, with five fields counted per duplicate totalling 10 fields counted per condition, error bars; ±95% CI, one-way anova. *P < 0.05.

To determine if NGF released from degenerating IVDs is contributing to the increase in CGRP immunoreactivity, experiments using an anti-NGF antibody were conducted. Like in PC12 cultures, 100 pg/ml of NGF was used as an additional control in this set of experiments. Following treatment with 100 pg/ml of NGF, 26 ± 1% of neurons were CGRP immunoreactive, which was not significantly different from 10 ng/ml NGF-treated neurons (*P* = 0.97). Incubating 100 pg/ml NGF media with normal IgG did not significantly alter CGRP immunoreactivity (*P* = 0.99). In contrast, incubating media containing 100 pg/ml of NGF with an anti-NGF antibody, CGRP expression was significantly reduced to 13 ± 3% (*P* = 0.038). Similarly, addition of anti-NGF antibody to degenerating, painful IVD media significantly reduced CGRP expression to 20 ± 2% (*P* = 0.048) when compared to degenerating media without the antibody (Fig.4, *n* = 2 IVD samples in each group).

### Degenerating IVDs release a multitude of pro-inflammatory and pro-nociceptive factors

**Figure 5:**
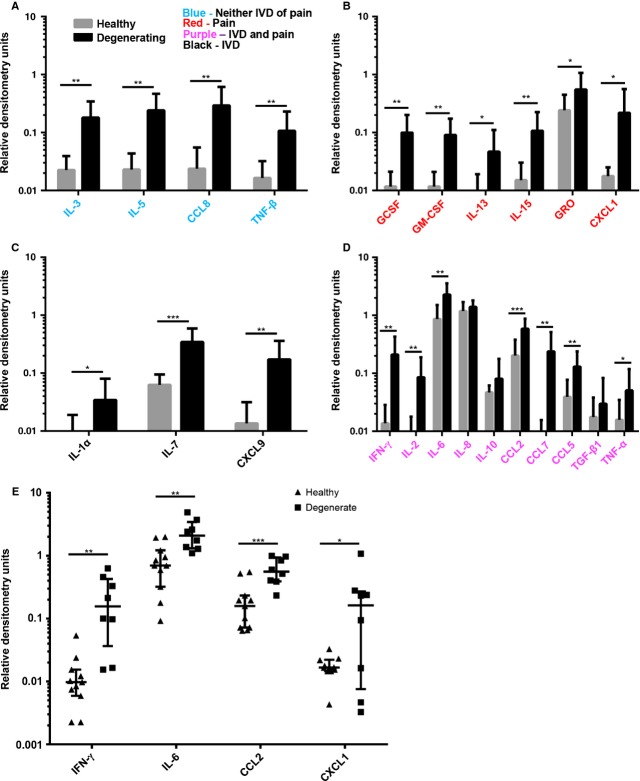
Comparison of factors released by healthy, pain-free and degenerating, painful intervertebral disc (IVDs) measured by protein arrays. The mean relative quantity of each factor released by healthy pain-free IVDs (grey bars) and degenerating, painful IVDs (black bars) are presented (A–D). Factors in blue have not been previously associated with disc degeneration or pain (A), factors in red have been associated with pain, but not disc degeneration (B), factors in black have been associated with disc degeneration (C) and factors in purple have been associated with both disc degeneration and pain (D). (E) Mean relative quantities of select factors involved in nociception are plotted to show individual variation between donors. *n* = 8 in degenerating, painful group and *n* = 11 in healthy, pain-free group, SEM for A–D, ±95% CI for E, unpaired *t*-test. *P < 0.05, **P < 0.01, ***P < 0.001.

Although the degenerating IVD media induced neurite growth, the same concentration range of NGF alone was insufficient. Therefore, protein arrays were used to identify potential cooperative factors [30]. Degenerating and painful IVDs released significantly higher levels of 20 of these factors (Fig.5A-D, Table1). Fifteen factors had a *P* value below 0.01 (GCSF, GM-CSF, IFN-γ, IL-2, IL-3, IL-5, IL-6, IL-7, IL-15, CCL2, CCL7, CCL8, MIG, RANTES and TNF-β), and five factors had a *P* value between 0.01 and 0.05 (IL-1α, IL-13, TNF-α, GRO and CXCL1). There was no difference in the relative quantities of IL-8, IL-10 and TGF-β1 between the two groups (Fig.5D, Table1). The relative mean quantities and a summary of previous studies implicating specific factors with either degenerating IVDs and/or pain are listed in Table1. Of particular interest are IFN-γ, IL-6, CCL2 and CXCL1 because of their suggested role in IVD degeneration, neuronal sensitization and pain [10,31–37]. Figure5E shows the individual donor variation in these factors. IFN-γ and CXCL1 showed a fairly large donor variation especially in the degenerate samples whereas the levels of IL-6 and CCL2 were much more homogeneous in their expression levels within each of the two groups.

**Table 1:** Comparison of 23 factors released by degenerating, painful or healthy pain-free IVDs

Factor	*P* value	Sig	Change	Relative mean densitometry units of healthy IVD media	Relative mean densitometry units of degenerating IVD media	IVD	Pain
GCSF	0.0025	**	Up	0.012 ± 0.003	0.1 ± 0.03		[50]
GM-CSF	0.0013	**	Up	0.012 ± 0.003	0.09 ± 0.03		[44]
IFN-γ	0.0022	**	Up	0.014 ± 0.004	0.21 ± 0.07	[12]	[33,34
IL-1α	0.0303	*	Up	0.0089 ± 0.003	0.034 ± 0.02	[15]	
IL-2	0.0069	**	Up	0.007 ± 0.003	0.085 ± 0.04	[12]	[45]
IL-3	0.0012	**	Up	0.023 ± 0.005	0.18 ± 0.05		
IL-5	0.0015	**	Up	0.023 ± 0.007	0.24 ± 0.08		
IL-6	0.0019	**	Up	0.87 ± 0.2	2.3 ± 0.4	[12,51	[36,44
IL-7	0.0002	***	Up	0.06 ± 0.01	0.34 ± 0.08	[52]	
IL-8	0.2434	N		1.2 ± 0.2	1.4 ± 0.1	[12]	[47]
IL-10	0.1169	N		0.047 ± 0.0046	0.081 ± 0.03	[51]	[11]
IL-13	0.0218	*	Up	0.0082 ± 0.002	0.047 ± 0.021		[48]
IL-15	0.0054	**	Up	0.015 ± 0.005	0.11 ± 0.04		[39]
CCL2	0.0005	***	Up	0.20 ± 0.05	0.59 ± 0.1	[12]	[11,30,37]
CCL8	0.0029	**	Up	0.024 ± 0.009	0.29 ± 0.1		
CCL7	0.0049	**	Up	0.007 ± 0.003	0.24 ± 0.09	[54]	[46]
CXCL9	0.0028	**	Up	0.014 ± 0.005	0.17 ± 0.06	[48]	
CCL5	0.0071	**	Up	0.039 ± 0.01	0.13 ± 0.04	[53]	[11,53
TGF-ß1	0.2251	N		0.018 ± 0.006	0.03 ± 0.02	[34,52	[49]
TNF-α	0.0473	*	Up	0.016 ± 0.0057	0.05 ± 0.02	[28,51	[13]
TNF-ß	0.0085	**	Up	0.017 ± 0.005	0.11 ± 0.04		
GRO	0.0286	*	Up	0.242 ± 0.07	0.55 ± 0.2		[30–32]
CXCL1	0.0304	*	Up	0.018 ± 0.002	0.22 ± 0.1		[30–32]

Relative mean densitometry unit quantities of 23 factors secreted by either healthy IVDs or degenerating, painful IVDs. Relative quantity of factors was analysed by RayBio Human Cytokine Array 1 Maps. *P* values were calculated using unpaired *t*-tests. The columns named, IVD and Pain, provide references describing previous studies implicating that factor with either degenerating IVDs and/or pain. Reviews were used when possible. *n* = 8 in degenerating, painful group and *n* = 11 in healthy, pain-free group, ±SEM, unpaired *t*-test.

* *P* < 0.05,

** *P* < 0.01,

*** *P* < 0.001.

## Discussion

Low back pain associated with IVD degeneration is a leading cause of chronic pain and morbidity, but how disc degeneration causes pain is not fully understood. Pain arises through a complex interplay between IVD matrix remodelling, the production of inflammatory, nociceptive and neurotrophic factors, nerve root and sensory neuron compression and disc innervation. Here, we show that degenerating IVDs surgically removed from axial low back pain patients release increased levels of several pro-inflammatory and pro-nociceptive factors that are able to drive neurite sprouting of PC12 cells and increase CGRP expression in primary neurons compared to healthy IVDs from donors who did not suffer from back pain. Moreover, we show that NGF is required to drive these observed effects. We demonstrate a direct link between disc degeneration and nociception by comparing degenerating and healthy human discs *ex vivo*.

Multiple studies have suggested that inflammatory and neurotrophic factors are present in degenerating disc tissue [8,29], and isolated IVD cells can actively secrete these factors in culture [27,28]. Moreover, treating cells isolated from either degenerating or healthy IVDs with cytokines, such as IL-1β or TNF-α, induces production of the neurotropic factors NGF and BDNF [38,39]. To establish a more direct link to *in vivo* disc degeneration, the present study uses a whole organ, *ex vivo* culture approach to compare the profile of factors released from degenerating and painful discs to healthy discs obtained from pain-free transplant donors. This approach leaves the IVD cells in their native environment and minimizes the effects of cell isolation and culture. In addition, the whole organ culture approach maintains *in vivo* cell densities whereas cell culture studies maintain much higher relative cell densities. These variables potentially affect IVD inter- and intracellular interactions, possibly influencing the profile of secreted factors.

While previous studies have implicated some of the factors in Figures1 and 5, Table1, NGF and BDNF in disc degeneration through *in vitro* cell culture studies or histological analysis, direct release of all of these factors has yet to be determined *in vivo*. Our *ex vivo* organ culture model demonstrates that degenerating IVDs from patients with chronic axial low back pain are releasing significantly higher levels of inflammatory and nociceptive factors compared to healthy discs. At this point it is not clear if all degenerating discs produce inflammatory and nociceptive factors or they are only produced by discs found in low back pain patients. We were unable to include degenerating IVDs from pain-free individuals in the present study because degenerating discs are not removed from individuals not suffering from chronic back pain and it is not possible to obtain a reliable long-term back pain history of an organ donor post-mortem. Many of these factors can potentially modulate neurite growth, nociceptive related neuroplasticity and chronic pain. These results suggest that inflammatory and nociceptive factors may be secreted *in vivo* from degenerating discs in low back pain patients, where they are likely playing a direct role in discogenic low back pain.

Nerve growth factor and BDNF are neuronal survival and growth factors. Previous studies demonstrated AF and NP cells isolated from degenerating human discs can increase neurite growth in co-cultures with the neuron-like SH-SY5Y cell line [16,40]. Among the factors identified in IVD conditioned media, degenerating and painful IVDs had elevated levels of NGF and BDNF. Surprisingly, the relatively low concentrations of these factors in the conditioned media significantly induced sprouting in PC12 cells. Anti-NGF treatment was sufficient to reduce neurite sprouting, demonstrating an important role for NGF in degenerating IVDs, even at low concentrations. However, when NGF is added to PC12 cultures at similar concentration to the one measured in degenerating IVD conditioned media, neurite sprouting was not induced. This data suggest that other factors released by degenerating IVDs are required in addition to NGF to induce neurite sprouting at concentrations below 100 pg/ml. Further studies are required to fully elucidate the mechanisms of painful, degenerating IVD media on neurite sprouting.

In addition to finding increased neurite growth, this study demonstrated that degenerating, painful IVDs secrete a combination of factors that increase CGRP expression in primary mouse DRG neurons. CGRP functions as a neurotransmitter and is strongly associated with pain. Calcitonin gene-related peptide expression can be increased by pro-nociceptive factors like NGF, TNF-α and CCL2 [10,37,41]. Understanding of nociceptor plasticity and CGRP changes associated with disc degeneration is mostly limited to animal models. Increased numbers of CGRP-immunoreactive neurons have been shown to innervate rat IVDs treated with complete Freund's adjuvant [42], a model for IVD inflammation. CGRP expression is also increased in a rat model of injury-induced IVD degeneration [43]. The present study demonstrates that degenerating and painful human IVDs also release factors that increase CGRP levels in neurons, thus further supporting the central hypothesis that degenerating and painful IVDs secrete factors known to contribute to nociception.

Nerve growth factor is a potent inducer of CGRP [10], and we therefore hypothesized that it mediates increased CGRP expression in cultures treated with degenerating, painful media. In contrast to previously published cell culture studies that use NGF concentrations higher than that found in disc media, we used a more similar concentration (Fig.1B) of 100 pg/ml. 100 pg/ml NGF was sufficient to increase CGRP expression to similar levels observed in cultures with 10 ng/ml NGF (Figs4B and 5B). This suggests that degenerating, painful IVDs secrete sufficient amounts of NGF to alter CGRP expression. Inhibiting NGF in degenerating disc media by incubation with an anti-NGF antibody caused a significant decrease in the percentage of CGRP-immunoreactive neurons compared to media without the antibody. This demonstrates that NGF found in media conditioned by degenerating, painful IVDs is sufficient to increase CGRP expression. This data suggest that NGF may play an important role *in vivo* in the development of chronic pain associated with intervertebral disc degeneration.

As 100 pg/ml of NGF is insufficient to drive neurite growth and other factors can sensitize neurons, we used protein arrays to identify additional factors that may play a role. Conditioned media from degenerating, painful IVDs and healthy, pain-free IVDs were analysed using cytokine and chemokine protein arrays. Twenty factors were found to be up-regulated in degenerating, painful samples, of which 10 (IFN-γ, IL-6 and -15, CCL2 and -7, CCL5, TNF-α, GRO and CXCL1, GCSF and GM-CSF) have been associated with increased nociception [10,11,31–37,44–50]. Some of the 20 factors have been previously associated with IVD degeneration [12,15,51–54], however 10 of the 20 factors (GCSF, GM-CSF, IL-3, IL-5, IL-13, IL-15, CCL8, TNF-β, GRO and CXCL1) have not previously been described in the IVD and two (CCL7 and CXCL9) [54] have not been described in degenerative disc disease. Of the 12 factors not described in pain associated with disc degeneration, seven (IL-13, IL-15, CCL7, GRO, CXCL1, GCSF and GM-CSF) have been associated with a variety of pain conditions (Fig.1, Table1). This data demonstrate that degenerating, painful IVDs secrete elevated levels of several pro-inflammatory and pro-nociceptive factors that are only secreted in very low basal levels by healthy, pain-free IVDs. As many of these factors have not been previously described in IVD degeneration; further investigation is warranted to understand their role in discogenic pain.

Multiple factors (including IFN-γ, IL-6, CCL2 and CXCL1, TNF-α, IL-1β, NGF and BDNF) known to be involved in nociception, development of chronic neuronal sensitization and hyperexcitability and chronic pain were increased in degenerating IVD conditioned media. For example, Robertson *et al*. have demonstrated that intrathecal injections of IFN-γ increase pain-related behaviour in mice [33] and Vikman *et al*. demonstrated that IFN-γ can induce increased excitability in dorsal horn neurons [34], suggesting that IFN-γ has a modulatory role in nociception. Similarly, in a rodent model of arthritis, IL-6 contributes to inflammatory pain, neuronal hyperexcitability and increased neuronal CGRP levels [35,36]. CCL2 can also increase CGRP production [30], and potentially acts as a pain-related neurotransmitter in DRG neuronal cultures [37]. CXCL1 has also been shown to induce increase nociceptor excitability and play an important role in inflammatory pain and neuronal sensitization [31,32]. While this study found inhibiting NGF was sufficient to inhibit degenerating disc induced CGRP increases, these other factors could contribute to the development of chronic low back pain through other mechanisms. However, further investigation of such mechanisms is required.

The present study shows that degenerating, painful IVDs secrete increased levels of multiple cytokines, chemokines and neurotrophins and that these factors increase neurite sprouting and CGRP expression. Furthermore, NGF secretion by degenerating, painful IVDs is sufficient to increase neurite sprouting and CGRP expression, which both can be blocked by anti-NGF antibody treatment. Taken together, this data suggest that factors actively released by degenerating and painful IVDs may induce innervation and pain *in vivo*. Furthermore, NGF may play an important role in nociception associated with IVD degeneration *in vivo*. Our data support further development of anti-NGF therapeutics to manage pain in degenerative disc disease [55–57]. A greater understanding of the molecular mechanisms driving pain associated with IVD degeneration may lead to improved therapies and quality of life for individuals with discogenic pain.
